# Association between obesity and ECG variables in children and adolescents: A cross-sectional study

**DOI:** 10.3892/etm.2013.1337

**Published:** 2013-10-09

**Authors:** GUO-ZHE SUN, YANG LI, XING-HU ZHOU, XIAO-FAN GUO, XIN-GANG ZHANG, LI-QIANG ZHENG, YUAN LI, YUN-DI JIAO, YING-XIAN SUN

**Affiliations:** 1 Department of Cardiovascular Medicine, The First Hospital of China Medical University, Shenyang, Liaoning 110001, P.R. China; 2 Library, Shengjing Hospital of China Medical University, Shenyang, Liaoning 110001, P.R. China

**Keywords:** obesity, abdominal obesity, electrocardiography, intervals, axes, children, adolescents

## Abstract

Obesity exhibits a wide variety of electrocardiogram (ECG) abnormalities in adults, which often lead to cardiovascular events. However, there is currently no evidence of an association between obesity and ECG variables in children and adolescents. The present study aimed to explore the associations between obesity and ECG intervals and axes in children and adolescents. A cross-sectional observational study of 5,556 students aged 5–18 years was performed. Anthropometric data, blood pressure and standard 12-lead ECGs were collected for each participant. ECG variables were measured manually based on the temporal alignment of simultaneous 12 leads using a CV200 ECG Work Station. Overweight and obese groups demonstrated significantly longer PR intervals, wider QRS durations and leftward shifts of frontal P-wave, QRS and T-wave axes, while the obese group also demonstrated significantly higher heart rates, compared with normal weight groups within normotensive or hypertensive subjects (P<0.05). Abdominal obesity was also associated with longer PR intervals, wider QRS duration and a leftward shift of frontal ECG axes compared with normal waist circumference (WC) within normotensive or hypertensive subjects (P<0.05). Gender was a possible factor affecting the ECG variables. Furthermore, the ECG variables, including PR interval, QRS duration and frontal P-wave, QRS and T-wave axes, were significantly linearly correlated with body mass index, WC and waist-to-height ratio adjusted for age, gender, ethnicity and blood pressure. However, there was no significant association between obesity and the corrected QT interval (P>0.05). The results of the current study indicate that in children and adolescents, general and abdominal obesity is associated with longer PR intervals, wider QRS duration and a leftward shift of frontal P-wave, QRS and T-wave axes, independent of age, gender, ethnicity and blood pressure.

## Introduction

Electrocardiogram (ECG) abnormalities are associated with an increased risk of adverse cardiovascular outcomes, including high resting heart rate (HR), prolonged PR interval, QRS duration and QT interval and abnormal shift in electrocardiographic axes (1). A prospective study has reported that resting HR has a significant positive association with cardiovascular and all-cause mortalities (2). A high HR is a strong indicator of cardiovascular mortality while a low HR is associated with an improved outcome (3). Prolonged PR interval has been associated with an increased risk of heart failure (HF), incident atrial fibrillation (AF), pacemaker implantation and mortality in the Framingham Heart Study (4,5). Prolonged PR interval is also correlated with endothelial dysfunction and activation of vascular repair, which may be a cause for adverse cardiovascular outcomes (6). QRS duration prolongation is a potential marker of cardiac structural and functional abnormalities, including left ventricular systolic dysfunction, that may predispose individuals to an increased risk of HF (7,8). A Framingham follow-up study demonstrated that healthy individuals with prolonged QRS duration were at a higher risk of future pacemaker implantation (9). Prolongation of corrected QT (QTc) interval predicts the risk for development of diabetes mellitus independently from conventional risk factors (10). It also predicts the risk of sudden death in patients without evidence of cardiac dysfunction (11). Electrocardiographic axes are also important markers in cardiovascular prognosis. For example, an abnormal P-wave axis is predictive of future onset of AF (12) and its verticalization is an effective electrocardiographic diagnostic tool for emphysema in the general population (13). Frontal QRS axis serves as a sign for left ventricular hypertrophy and bundle branch block. The T axis is a general marker of repolarization abnormality. A follow-up study on older patients revealed that T-wave axis deviation is a strong independent risk indicator of fatal and non-fatal cardiac events (14). Therefore, it is necessary to investigate factors affecting or causing these electrocardiographic changes.

Obesity is a strong independent risk factor for cardiovascular disease mortality and it predisposes patients to numerous cardiac complications, including hypertension, coronary heart disease, HF, stroke and sudden death (15,16). The association between obesity and ECG has been investigated in previous studies. Obesity is closely associated with a wide variety of ECG abnormalities, including ischemic ECG observations (17), leftward shifts in electrocardiographic axes, markers of left ventricular hypertrophy and flattening of the T wave (18). High resting HR and prolongation of PR interval and QRS duration are changes in electrocardiographic intervals induced by obesity (19–21). A number of these ECG alterations may be reversed through weight loss, including rightward shift of the mean P-wave, QRS and T-wave axes (22).

Numerous studies have reported a correlation between obesity and QTc interval prolongation (20,21); however, one study showed no correlation between body mass index (BMI) and QTc interval in a healthy population aged 22–25 years (23). Similarly, the effect of obesity on QTc interval in children is also controversial (24,25). Furthermore, ECG has been associated with age, gender, ethnicity and blood pressure (26,27). However, the subjects in the majority of the previous studies were adults while the correlation between obesity and ECG in children and adolescents has not been studied in detail on a large-scale. An additional concern is that waist circumference (WC) and waist-to-height ratio (WHtR), measures of abdominal fat distribution, are more efficient risk factor predictors of cardiovascular disease than BMI in children (28). However, there have been no studies to date concerning the correlations between body fat distribution and ECG in children and adolescents.

Therefore, in this study, the differences in electrocardiographic intervals and axes associated with various degrees of obesity in normotensive and hypertensive children and adolescents were examined in a large-scale population. The final aims were to determine the effect of obesity on electrocardiographic variables besides the aforementioned possible risk factors and identify the possible association with body fat distribution in children and adolescents.

## Materials and methods

### Study protocol

The study protocol and the procedures were approved by the Ethics Committee of China Medical University (Shenyang, China) and fully informed consent was obtained from the parents or legal guardians of all subjects.

### Study population

A total of 5,556 Chinese elementary and secondary school students aged 5–18 years were recruited from 12 rural public schools using multistage cluster sampling in this cross-sectional study between July 2010 and January 2011 in Shenyang (China). The following exclusion criteria and processes were enforced: i) 28 students who had any history of heart diseases, diabetes mellitus, chronic renal diseases or thyroid diseases were excluded; ii) 91 students who had abnormal ECGs, including atrial rhythm, premature beat, ventricular tachycardia, pre-excitation wave, right bundle branch block or apparent first degree atrioventricular block, were excluded. Therefore, the final study group consisted of 5,437 students (2,952 males and 2,485 females). Clinical data (age, gender, ethnicity, height, weight, WC and blood pressure) and standard 12-lead ECG was collected for each participant in this study.

### Anthropometric measurements

Body weight, height and WC measurements were all performed by well-trained personnel (cardiologists, doctors of internal medicine and pediatricians) using the standard protocols. Body weight and height were measured while the participants were barefoot and in light underclothes, to the nearest 0.1 kg and 0.5 cm, respectively. WC was measured to the nearest 0.5 cm at umbilicus level following a normal expiration using a non-elastic tape (29). BMI was calculated using the following formula: Weight (kg)/height^2^ (m^2^). Overweight and obesity were defined by the recommended BMI cutoff values (through BMI of 25 and 30 kg/m^2^ at 18 years of age) by the International Obesity Task Force, according to age and gender (30). The age and gender-specific 90th percentile WC cutoffs newly developed by the Working Group on Obesity in China were used to define central obesity (31).

### Blood pressure measurements

Blood pressure was measured in all subjects in this study by well-trained personnel using a mercury sphygmomanometer and appropriate size cuffs. The subjects were advised to avoid coffee, tea and exercise for ≥30 min and had rested for ≥5 min prior to the measurement. Systolic blood pressure (SBP) and diastolic blood pressure (DBP) were measured twice in the right arm in the supine position with the arm level with the heart. The average of the two blood pressure measurements, SBP and DBP, was used in the analysis. According to the Fourth Report on the Diagnosis, Evaluation and Treatment of High Blood Pressure in Children and Adolescents, hypertension was defined as having SBP and/or DBP levels above the 95th percentile for gender, age and height (32).

### ECG variable measurements

Standard simultaneous 12-lead resting ECGs were recorded for participants in a supine position at a 1,000-Hz sampling rate with the use of a PC-based acquisition and analysis system (CV200 ECG Work Station; Vales and Hills Biomedical Tech. Ltd., Beijing, China) and stored in a personal computer as separate files for subsequent processing. All recordings were performed by well-trained technicians and all digital ECGs were measured and interpreted on the computer by the same well-trained cardiologist. Cases with apparent disturbances or lack of one or more leads were excluded from the study population. In the CV200 ECG Work Station, temporally aligned superimposed ECG leads, at a magnification of five-fold for paper speed (125 mm/sec) and four-fold for amplitude (40 mm/mV), were made available in order to facilitate the measurement of variables and to validate the onset and end points of the intervals, in accordance with the American Heart Association/American College of Cardiology/Heart Rhythm Society (AHA/ACCF/HRS) recommendations (33). The key points were identified manually and then the values of intervals and frontal axes were calculated automatically by the computer. Detailed definitions of each interval in this study were as follows: HR, calculated by a computer, average heart beats within 20 sec; PR interval, interval from the earliest onset of P wave to the earliest onset of QRS complex among 12 leads; QRS duration, interval from the earliest onset to the latest offset of QRS complex among 12 leads; QT interval, interval from the earliest onset of the QRS complex to the latest offset of the T wave among 12 leads; and QTc interval, calculated using Bazett’s formula: QTc = QT/(RR interval)^1/2^.

### Statistical analysis

Continuous variables are expressed as mean ± standard deviation (SD). Categorical data are presented as frequencies and percentages. Differences between the groups were compared using the two-tailed, non-paired Student’s t-test or one-way analysis of variance for continuous variables, where appropriate. Comparisons of categorical variables between groups were performed using the χ^2^ test. Pearson’s correlation coefficients were used to explore the correlation between ECG variables and obesity-associated measurements, including BMI, WC and WHtR. The linear associations between variables were further examined using the multivariate linear regression analysis. All tests of statistical significance were two-sided and P<0.05 was considered to indicate a statistically significant difference. The statistical analyses were conducted with SPSS 17.0 (SPSS, Inc., Chicago, IL, USA).

## Results

### Demographic and clinical characteristics

The final study sample consisted of 5,437 participants (2,952 males and 2,485 females) with a mean age of 10.9±2.7 years. Of the study population, 16.8% were presented as overweight (male, 18.4%; female, 14.9%) and 7.3% as obese (male, 10.2%; female, 3.9%). Abdominal obesity was found in 502 males (17.0%) and 339 females (13.6%) with an overall prevalence of 15.5%. The prevalence of abdominal obesity increased significantly with BMI categories (normal weight, 1.9%; overweight, 43.8%; obesity, 90.7%).

The baseline characteristics of the participants enrolled in this study according to BMI category are presented in Table I. The mean ages among the three groups were similar. However, the constituent ratio of the male subjects was significantly higher than those of the female subjects in the overweight and obese group with the male subject ratio at 59.5 and 75.6%, respectively. Among the participants, an increased ratio of Manchu ethnicity and a decreased ratio of Han ethnicity with BMI categories were also present. Body weight, height, WC and WHtR were positively associated with the degree of overall obesity. The mean levels of SBP and DBP in the overweight or obese groups were significantly higher than the normal weight group and increased with BMI categories.

### Differences in ECG variables associated with overall obesity

The differences in electrocardiographic intervals and axes among normal weight, overweight and obese groups are listed in Table II. The mean levels of variables are presented for various weight groups and stratified by blood pressure. Compared with the normal weight group, longer PR interval, wider QRS duration and leftward shift of frontal QRS axis were apparent in the overweight and obese groups within normotensive and hypertensive subjects. Obese participants had a 4.2-msec longer PR interval, 4.0-msec wider QRS duration and 9.6° leftward shift of frontal QRS axis than the normal weight group within normotensive subjects, while the differences within hypertension subjects were 4.2 msec, 4.8 msec and 10.0°, respectively. A higher HR, with an average increase of 3.1 beats/min, existed in the obese group compared with normal weight within normotensive subjects while the difference among the three groups within hypertensive subjects was not significant. The frontal P-wave axis presented a leftward shift trend along with aggravation of obesity, but the difference was significant only between obese and normal weight groups within hypertensive subjects. A significant leftward shift in the frontal T-wave axis was presented in the overweight and obese groups compared with the normal weight group within normotensive subjects; while a significant change was presented only in the obese group within hypertensive subjects. However, no significant differences were noted in the QTc interval among the three groups within normotensive or hypertensive subjects.

**Figure 1:**
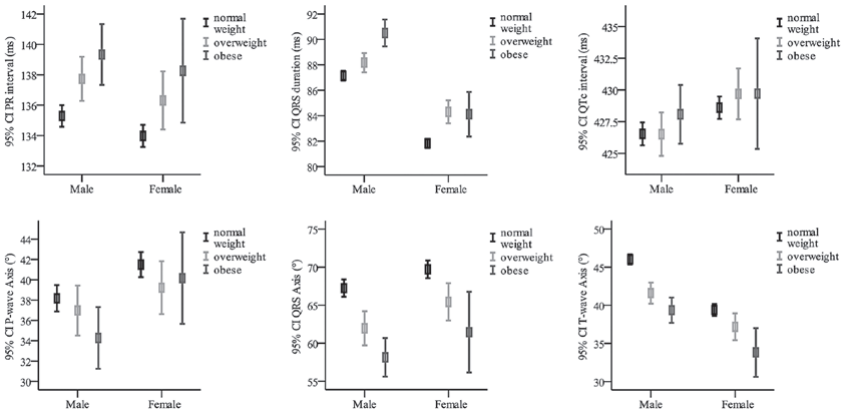
Gender-stratified analysis of the associations between obesity and electrocardiogram (ECG) variables.

Furthermore, detailed differences of electrocardiographic intervals and axes among normal weight, overweight and obese groups stratified by gender are presented in Fig. 1. The results from males and females showed similar trends in ECG changes associated with obesity, and gender itself was a factor affecting ECG variables.

### Differences in ECG variables associated with abdominal obesity

The differences in electrocardiographic intervals and axes between abdominal obesity and normal WC, stratified by blood pressure, are listed in Table III. Compared with the normal WC group, the abdominal obese group had significantly longer PR interval, wider QRS duration, leftward shifts of frontal P-wave axis and QRS axis. The differences in normotensive subjects aforementioned were 3.9 msec, 2.1 msec, 2.8° and 7.4°, respectively. These differences were larger in hypertensive subjects with values of 5.5 msec, 3.5 msec, 4.4° and 8.2°, respectively. The frontal T-wave axis showed 5.2° leftward shift in the abdominal obese group compared with the normal WC group in normotensive subjects (P<0.001); however, the difference in hypertensive subjects was not significant (38.8±15.4 versus 40.9±17.4; P=0.056). No significant differences were noted in HR and QTc interval between the two groups in normotensive or hypertensive subjects.

### Correlations with BMI, WC and WHtR

Pearson correlation coefficients between ECG variables and measures of obesity are presented in Table IV. Although the correlation coefficients decreased following adjustment for age, gender, ethnicity, SBP and DBP, electrocardiographic intervals, including PR interval and QRS duration, still correlated positively with the three measures of obesity (PR interval, BMI r=0.076, WC r=0.081, WHtR r=0.043, each P<0.001; QRS duration, BMI r=0.101, WC r=0.099, WHtR r=0.063, each P<0.001). Despite the fact that the associations between P-wave axis, BMI and WC prior to adjustment were not significant, electrocardiographic axes, including P-wave, QRS and T-wave axes correlated negatively with measures of obesity following adjustment (P-wave axis, BMI r=−0.048, WC r=−0.062, WHtR r=−0.067, each P≤0.001; QRS axis, BMI r=−0.103, WC r=−0.097, WHtR r=−0.103, each P<0.001; T-wave axis, BMI r=−0.125, WC r=−0.116, WHtR r=−0.124, each P<0.001). No significant correlation was found with BMI or WC in HR and QTc intervals. HR correlated positively with WHtR (r=0.029; P<0.05).

Considering differences in baseline characteristics among various weight groups may have possible impacts on the study results, multivariate linear regression analysis was performed to explore the linear association between ECG variables and obesity. Multivariate linear regression data are listed in Table V.

## Discussion

The prevalence of overweight and obese children is a growing global health concern according to the World Health Organization (34). A previous study indicated that WC and WHtR, measures of abdominal obesity, were more efficient risk factor predictors of cardiovascular disease than BMI in children (28). In the present study, the prevalence of general obesity and abdominal obesity in children and adolescents was reported, using the recommended BMI and WC percentiles. It was found that being overweight and obese is common among rural Chinese children, with 16.8% overweight and 7.3% obese. Comparing the collected data with the prevalence rate of obesity among urban Chinese children, based on the data published by the sixth Chinese National Survey on Students Constitution and Health in 2010 (35), a slightly lower prevalence (0.8%) in obesity among rural Chinese individuals was observed. Higher prevalence of obesity in males was also found, in accordance with the sixth Chinese National Survey. The overall prevalence of abdominal obesity among the rural public school subjects was 15.5% and similar gender differences were observed with higher prevalence in males than females (17.0 versus 13.6%).

Obesity in adults is closely associated with a wide variety of ECG changes, including high resting HR, prolongation of PR interval and QRS duration and leftward shifts of electrocardiographic axes. However, the effect of obesity on QTc intervals is controversial in children and adults. Significant positive associations between BMI and ECG intervals, including PR interval and QRS duration, were reported in this cross-sectional study. Furthermore, these associations were linear and independent of age, gender, ethnicity and blood pressure (20). Significant negative associations were noted between BMI and electrocardiographic axes (P-wave, QRS and T-wave) in the results, which were consistent with previous studies (18,20). The difference in HR between the normal weight and obese groups within normotensive subjects was significant. However, no significant differences existed within hypertensive subjects and the correlation between HR and BMI was also insignificant following adjustment for age, gender, ethnicity, SBP and DBP. The results were inconsistent with previous studies (19,20,36), which may be due to the effect of other factors. The results in the present study showed that the QTc interval was not associated with obesity which was similar to the result by Leotta *et al* in a healthy young population (23).

Abdominal obesity is an independent cardiovascular risk factor; however, the association between abdominal obesity and ECG variables has not been examined previously in large-scale populations. Differences in ECG intervals and axes between normal WC and abdominal obesity in rural Chinese children and adolescents were explored in this study. Abdominal obesity was associated with longer PR intervals, wider QRS duration and leftward shifts in frontal P-wave and QRS axes compared with the normal WC. Leftward shift of frontal T-wave axis was also apparent, although the difference within hypertensive participants was not significant. The ECG variables, including PR interval, QRS duration, frontal P-wave axis, QRS axis and T-wave axis, correlated with WC and WHtR following adjustment. Furthermore, these associations were linear and independent of age, gender, ethnicity and blood pressure. In general, the effects of abdominal obesity on ECG were in agreement with general obesity.

In measuring ECG values, this study differed in two ways from the others. Firstly, a combination of manual locations and computer-based calculations was used in the evaluation of the ECG variables, as described previously, while the majority of previous studies used computer-generated calculations. Manual operation may reduce mistakes in measurements made by the computer (37). Secondly, the measurements were based on the simultaneously recorded 12-lead ECGs and temporally aligned superimposed ECG leads were made available as an optional display under the CV200 ECG Work Station to validate the onset and end points of the intervals according to the AHA/ACCF/HRS recommendations (38). The measurements in the majority of previous studies were based on other ECG leads.

There were certain limitations to the present study. As a cross-sectional study, the associations between obesity and ECG intervals and axes were explored; however, the long-term interaction between obesity and ECG variables was not elucidated. Furthermore, the electrocardiographic changes were relatively small and all within normal limits. Therefore, whether these differences had clinical significance was unclear. These issues require more investigation and further follow-up studies.

In conclusion, ECG variables were examined and a novel result was reported from a large-scale cross-sectional study among children and adolescents in rural Liaoning in Northeast China. In addition, general and abdominal obesity in children and adolescents was revealed to be associated with longer PR intervals, wider QRS duration and leftward shifts in frontal P-wave, QRS and T-wave axes. Gender itself was a possible factor affecting the ECG variables. In addition, these associations with BMI, WC and WHtR were linear and independent of age, gender, ethnicity and blood pressure.

## Figures and Tables

**Table I:** Demographic, anthropometric and blood pressure characteristics of the study sample, classified by BMI.

Variables	Normal weight (n=4,126)	Overweight (n=913)	Obese (n=398)	P-value
Age, years	10.9±2.7	10.8±2.7	10.7±2.7	0.202
Male gender, n (%)	2,108 (51.1)	543 (59.5)	301 (75.6)	<0.001
Han/Manchu/other ethnicity, %	82.1/9.8/8.1	80.6/11.0/8.4	76.2/15.2/8.6	0.033
Weight, kg	36.7±10.9	48.7±14.8	61.5±19.5	<0.001
Height, cm	143.1±15.1	145.5±15.2	147.7±16.0	<0.001
WC, cm	59.0±7.5	70.8±10.1	82.8±12.4	<0.001
WHtR	0.413±0.035	0.486±0.039	0.560±0.049	<0.001
BMI, kg/m^2^	17.4±2.1	22.3±2.6	27.3±3.8	<0.001
SBP, mmHg	106.8±12.6	112.9±14.6	119.2±15.3	<0.001
DBP, mmHg	65.9±9.7	68.7±11.0	71.6±10.5	<0.001
Abdominal obesity, n (%)	80 (1.9)	400 (43.8)	361 (90.7)	<0.001

WC, waist circumference; WHtR, waist-to-height ratio; BMI, body mass index; SBP, systolic blood pressure; DBP, diastolic blood pressure.

**Table II:** Differences in electrocardiographic intervals and axes among overall weight groups by BMI, stratified by blood pressure.

	Normotension (n=4,341)				Hypertension (n=1,096)			
		
Variables	Normal weight (n=3,491)	Overweight (n=641)	Obese (n=209)	P-value	Normal weight (n=635)	Overweight (n=272)	Obese (n=189)	P-value
HR, beats/min	85.2±13.2	85.9±13.8	88.3±13.1^b,c^	0.002	88.2±13.9	89.1±14.6	86.8±13.8	0.240
PR interval, msec	134.5±16.3	136.8±17.8^a^	138.7±17.5^b^	<0.001	135.3±17.9	138.0±17.8^a^	139.5±17.4^b^	0.007
QRS duration, msec	84.5±8.9	86.3±9.1^a^	88.5±9.3^b,c^	<0.001	84.7±9.6	87.4±9.2^a^	89.5±9.9^b,c^	<0.001
QTc interval, msec	427.2±20.7	426.9±20.0	427.8±21.5	0.862	429.3±21.0	429.9±20.1	429.2±19.7	0.923
P-wave axis, °	39.4±29.4	37.1±26.7	36.5±25.3	0.084	42.2±30.2	39.9±29.9	34.8±26.5^b^	0.010
QRS axis, °	68.3±26.8	64.0±25.5^a^	58.7±23.7^b,c^	<0.001	69.2±25.7	61.9±25.6^a^	59.2±23.0^b^	<0.001
T-wave axis, °	43.0±16.8	40.2±17.0^a^	37.4±16.1^b,c^	<0.001	41.3±17.8	38.9±16.3	38.7±13.8^b^	<0.050

^a^P<0.05 and ^b^P<0.05, vs. normal; ^c^P<0.05, vs. overweight. BMI, body mass index; HR, heart rate; QTc, corrected QT.

**Table III:** Differences in electrocardiographic intervals and axes between abdominal obese and normal WC group, stratified by blood pressure.

	Normotensive (n=4,341)			Hypertensive (n=1,096)		
		
Variables	Normal WC (n=3,822)	Abdominal obese (n=519)	P-value	Normal WC (n=744)	Abdominal obese (n=322)	P-value
HR, beats/min	85.4±13.3	85.9±13.3	0.433	88.1±14.0	88.4±14.3	0.809
PR interval, msec	134.6±16.5	138.5±17.2	<0.001	135.1±17.7	140.6±17.8	<0.001
QRS duration, msec	84.7±9.0	86.8±9.2	<0.001	85.1±9.4	88.6±10.1	<0.001
QTc interval, msec	427.3±20.6	426.3±20.5	0.263	429.3±20.7	429.8±20.2	0.693
P-wave axis, °	39.2±29.2	36.4±26.1	0.023	41.6±29.2	37.2±30.3	0.025
QRS axis, °	68.1±26.7	60.7±25.2	<0.001	68.1±25.2	59.9±25.5	<0.001
T-wave axis, °	43.0±16.7	37.8±17.4	<0.001	40.9±17.4	38.8±15.4	0.056

WC, waist circumference; HR, heart rate; QTc interval, corrected QT interval.

**Table IV:** Pearson’s correlation coefficients between electrocardiographic variables and BMI, WC and WHtR.

	BMI		BMI^a^		WC		WC^a^		WHtR		WHtR^a^	
						
Variables	r	P-value	r	P-value	r	P-value	r	P-value	r	P-value	r	P-value
HR	−0.037	0.006	0.028	0.061	−0.080	<0.001	0.012	0.398	0.031	0.024	0.029	0.049
PR interval	0.160	<0.001	0.076	<0.001	0.185	<0.001	0.081	<0.001	0.057	<0.001	0.043	0.003
QRS duration	0.161	<0.001	0.101	<0.001	0.174	<0.001	0.099	<0.001	0.127	<0.001	0.063	<0.001
QTc duration	0.011	0.409	−0.002	0.897	0.004	0.771	−0.012	0.432	−0.016	0.231	−0.015	0.317
P-wave axis	−0.013	0.331	−0.048	0.001	−0.014	0.296	−0.062	<0.001	−0.067	<0.001	−0.067	<0.001
QRS axis	−0.119	<0.001	−0.103	<0.001	−0.117	<0.001	−0.097	<0.001	−0.119	<0.001	−0.103	<0.001
T-wave axis	−0.106	<0.001	−0.125	<0.001	−0.088	<0.001	−0.116	<0.001	−0.091	<0.001	−0.124	<0.001

a Adjusted for age, gender, ethnicity, systolic blood pressure and diastolic blood pressure.

r, Pearson’s correlation coefficient; BMI, body mass index; WC, waist circumference; WHtR, waist-to-height ratio; HR, heart rate; QTc interval, corrected QT interval.

**Table V:** Multivariate linear regression analysis for the association between body habitus and electrocardiographic intervals and axes.

	BMI		WC		WHtR	
			
Variables	β (95% CI)	P-value	β (95% CI)	P-value	β (95% CI)	P-value
HR	0.112 (−0.005, 0.228)	0.061	0.019 (−0.025, 0.062)	0.398	6.959 (0.038, 13.880)	0.049
PR interval	0.390 (0.243, 0.537)	<0.001	0.155 (0.100, 0.210)	<0.001	13.024 (4.286, 21.762)	0.003
QRS duration	0.273 (0.195, 0.350)	<0.001	0.099 (0.070, 0.128)	<0.001	10.183 (5.567, 14.799)	<0.001
QTc duration	−0.012 (−0.195, 0.170)	0.897	−0.027 (−0.095, 0.041)	0.432	−5.523 (−16.354, 5.308)	0.317
P-wave axis	−0.430 (−0.689, −0.172)	0.001	−0.208 (−0.304, −0.112)	<0.001	−35.743 (−51.080, −20.407)	<0.001
QRS axis	−0.842 (−1.076, −0.608)	<0.001	−0.296 (−0.383, −0.209)	<0.001	−50.098 (−63.991, −36.205)	<0.001
T-wave axis	−0.649 (−0.797, −0.501)	<0.001	−0.224 (−0.279, −0.169)	<0.001	−38.230 (−47.011, −29.448)	<0.001

Multivariate analysis was adjusted for age, gender, ethnicity, systolic blood pressure and diastolic blood pressure. β, unstandardized regression coefficients; BMI, body mass index; WC, waist circumference; WHtR, waist-to-height ratio; HR, heart rate; QTc interval, corrected QT interval; CI, confidence interval.

## Abbreviations

ECG: electrocardiography
HR: heart rate
AF: atrial fibrillation
HF: heart failure
QTc: corrected QT interval
CHD: coronary heart disease
BMI: body mass index
WC: waist circumference
WHtR: waist-to-height ratio
SBP: systolic blood pressure
DBP: diastolic blood pressure
